# Transformations of phenolic compounds in meadow soils

**DOI:** 10.1038/s41598-020-76316-7

**Published:** 2020-11-09

**Authors:** Anna Ziolkowska, Bozena Debska, Magdalena Banach-Szott

**Affiliations:** Department of Environmental Chemistry, University of Science and Technology, 6 Bernardynska St, 85-029 Bydgoszcz, Poland

**Keywords:** Environmental sciences, Chemistry

## Abstract

The aim of the research has been to determine the role of phenolic compounds in the processes of transformations of organic matter in meadow soils, leading to the formation of humic substances. The research has been performed based on the plant material and soil sampled from Europe’s unique complex of permanent grasslands irrigated continuously for 150 years applying the slope-and-flooding system, the Czerskie Meadows. Phenolic compounds were isolated from the plant material samples (hay, sward and roots) and soils (horizon A, AE and Bsv) and from the fraction of humic and fulvic acids. It was found that the contents of phenolic compounds decrease in the following order: hay > sward > roots > A horizon soil > AE horizon soil > Bsv horizon soil > A horizon fulvic acids > AE horizon fulvic acids > Bsv horizon fulvic acids > A horizon fulvic acids > AE horizon fulvic acids > Bsv horizon fulvic acids. A significantly higher share of cinnamyl than vanillyl and syringyl compounds in the extracts of fulvic acids and slightly higher in the hydrolysates of humic acids confirms the effect of the chemical composition of the plant material undergoing decomposition on the properties of the emerging humic substances.

## Introduction

The investigation of the state of vegetation and soils as well as their protection is an essential aspect at global, regional and local scale, which, at the same time, is one of the key objectives of the European Community policy. The Kyoto Protocol provides that soil, thanks to its process of sequestration, is the biggest storage of carbon resources to be protected and increased as much as possible^1–8^.

The importance of meadow soils is reflected in a higher content of organic substance as compared with arable soils. Meadow soils provide better conditions for the accumulation and immobilization of organic substance. At the same time the grasslands are considered to use the land in a way which is favourable to organic carbon sequestration in soil^8^.

Organic matter in meadow soils comes mostly from dead plant roots, found-on-the-surface plant residue and dead microorganisms. The permanent grassland vegetation is made of perennial species, and the key role is played by grasses and the *Fabaceae*. In hay meadows, grasses account for 60–80% of the share in the sward, while the *Fabaceae* – for 5–10%, the rest are herbs and weeds, and their share depends on the level of nursing^9^.

The key compounds of the plant cell walls are cellulose, hemicelluloses and lignins, the compounds playing an essential part in the processes of humification. The content of lignins increases with the development of graminaceous plants, and their chemical composition depends on the kind of plant tissues and their anatomy. Karczmarczyk^10^, Lindedam et al.^11^ and Meier et al.^12^ show that in grass communities the key source of lignins are roots (11–20% d.w.). In the aboveground part of arable grasses, the content of lignins depending on the plant organ and development stage accounts for 2.1–9.1% d.w.

The precursors of humic substances can be also tannins, terpenes, metabolites of microorganisms and the products of autolysis of their cells. Irrespective of the theory of the humic substances formation in soils, it is unquestionable that the key role in the process of humification is played by phenolic compounds. The compounds are one of the most common components of soils^13,14^and they affect the cycle of the key nutrients for plants and soil microorganisms^14,15^. Phenolic compounds in soils can occur in a dissolved form, sorbed form, and a polymerized form. Despite the numerous phenol studies, there has been still some controversy e.g. in terms of phenol transformations in soil and the effect on the decomposition rate of soil organic matter. Interestingly, some authors suggest that the form of phenols (dissolved, sorbed and polymerized), and not their chemical structure, can affect their presence in soils^16–19^. For example, dissolved phenols can have a greater chance to get transformed into simple and available forms by microorganisms in the soil solution. On the other hand, physically and chemically protected phenolics can persist longer than dissolved forms, providing feedbacks to SOM decomposing microorganisms via changing soil pH, nutrient availability, and enzyme activities.

The environmental factors which can affect the degradation of phenols include the soil pH, temperature, moisture and the availability of oxygen. Sinsabaugh^20^ found that there is a positive correlation between phenolics degrading enzyme activities and soil pH across ecosystems. Similar dependencies have been reported by Pind et al.^21^ in peat soils. According to^22–24^, the activity of phenol oxidase did not show any clear dependence on temperature in field conditions probably due to the interactive effect of oxygen availability at different temperatures. The dependence between the activity of phenolic oxidase and the concentration of phenols in the natural ecosystem remains ambiguous. According to some authors, the correlation is positive^25–27^, and according to others – negative^28,29^, and so predicting the direction of phenolics processing may be difficult. As for the soils with a high amount of phenols, e.g. peats, higher phenol oxidase in soil results in a higher phenolic content in pore water as a product of enzyme action on peat, resulting in a positive correlation between phenol oxidase and phenolics. Most studies report on drought increasing the activity of phenol oxidase, thus increasing the intensity of decomposition^30–32^.

The production of phenols from plant residue, its delayed degradation in soil and SOM decomposition also depend on the level of CO_2_. Elevated CO_2_ increased DOC and phenolic leaching from wetlands^33,34^, which may decrease hydrolase activities^25^.

Unlike the rather unidirectional effects of elevated CO_2_, warming has various effects on the production of phenolics. The increases in temperature have led to both an increase and a decrease in phenolic production^35^.

Phenolic compounds are a component of lignins and they can be produced as a result microbiological biosynthesis from aliphatic substrates^5,9,36–38^. Changes in the content of lignins and the level of their transformation in decomposing material can be evaluated with the oxidation of plant material and soil samples by using CuO, or applying the method of acid or/and alkaline hydrolysis^36,39–44^. With those methods it is possible to release aldehydes and phenolic acids from lignins. Identifying phenolic compounds allows for separating the groups referred to as:

- vanillyl (V) compounds, as the total content of vanillyl aldehyde and vanillyl acid, derived from coniferyl alcohol;
- syringyl (S), as the total content of syringyl acids and aldehyde, derived from sinapic alcohol;
- cinnamyl (C), as the total contents of ferulic, *p*-coumaric acid and caffeic acids, derived from coumaryl alcohol.

As demonstrated by e.g. Kovaleva and Kovalev^37^ and Crawford^45^, the ratios of those compounds in lignins are plant species specific. The lignins of flowering plants contain approximately equal amounts of vanillyl and syringyl compounds and little cinnamyl compounds (V:S:C is approximately 49:46:5). The gymnosperms lignins show a definite advantage of vanillyl compounds (V:S:C – 80:6:14), whereas the lignins of herbs contain higher amounts of cinnamyl compounds, as compared with the content of vanillyl and syringyl compounds. The lignins of meadow grasses and herbs can contain 4- to 6-fold more cinnamyl compounds than the lignins of trees. Kovaleva and Kovalev^37^ recorded for the aboveground part of subalpine meadows graminaceous plants the V:S:C value of 1:1:6.

The structure of the lignin fraction derived from the plant material gets considerably transformed in soils during humification. Banach-Szott and Debska^39,40^ stress that changes in the content and ratios of those compounds facilitate determining the degree of plant material humification. Kovalev and Kovaleva^36^, Sjoberg^44^ have also indicated that an important parameter being an indicator of the degree of lignin decomposition, as a measure of the amount of undisturbed uncondensed lignin structures, is the total content of vanillyl, syringyl and cinnamyl compounds (V + S + C).

As seen from the research by Kovaleva and Kovalev^37^ and Banach-Szott and Debska^39,40^, phenolic compounds are also present in the fractions of humic and fulvic acids (FAs). In general, humus acids show a similar qualitative composition of lignin oxidation products as compared with soil hydrolysates^37^. Banach-Szott and Debska^39^ have demonstrated that the value of parameter V + S + C in the fractions of humus acids decreases with an increase in the degree of humification of the plant material and fulvic acids contain more phenolic compounds, as compared with the extracts of the humic acids (HAs) fraction. As reported by Kovaleva and Kovalev^37^ in meadows with herbs prevailing, the ratio of vanillyl, syringyl and cinnamyl compounds for humic and fulvic acids is 1:1:1, whereas for the fraction of humus acids isolated from forest soils, the occurrence of definite shares of vanillyl compounds (as much as up to 2:1:1 and 5:1:1) is characteristic.

Kovaleva and Kovalev^37^, Banach-Szott and Debska^40^ and Sjoberg et al.^44^ have indicated that the parameter which can be also used to evaluate the degree of lignin decomposition is the ratio of the content of vanillyl acid to the content of vanillyl aldehyde. During the plant material decomposition, vanillyl aldehyde gets oxidised to vanillyl acid. For humic substances of the soils of subalpine meadows the value of that ratio in the soil layer at the depth of 5–35 cm was 1.60, while at the depth of 35–57 cm – 6.20^37^.

Interestingly, it is very sporadic that one finds papers^37^ at the same time presenting the results of the research of the content of phenolic compounds found in plant material, soils and in the fractions of humic substances, and only such research can provide information on the importance of the plant material in the processes of humic substances formation (humification processes).

With the above in mind, in the paper a hypothesis has been assumed that identifying phenolic compounds and determining their content in plant material, soils and in the fractions of humic and fulvic acids can facilitate determining the transformations of the plant material of meadow soils and thus enhance a further development of the knowledge on the course of humification processes.

The aim of the study has been to determine the role of phenolic compounds in the processes of meadow soil transformations leading to the formation of humic substances.

## Materials and methods

### Materials

**Figure 1:**
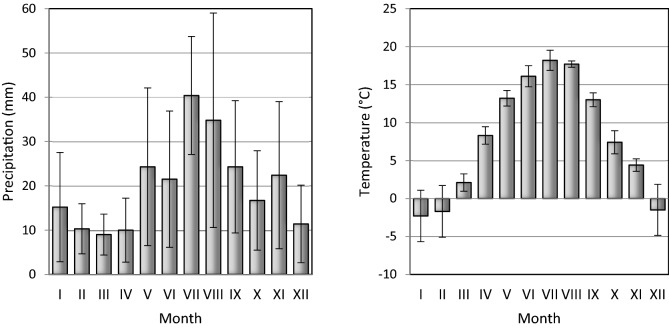
(**a)** Variation in precipitation and (**b)** mean annual temperature in 2008–2012.

The research was performed in Europe’s unique complex of perennial grasslands irrigated continuously for about 150 years applying the slope-and-flood system; the Czerskie Meadows, in the past managed by a meadow farming company known as the Board of Royal Meadows to provide the farm animals with the required amount of feed and thus to limit the people emigrating from those areas^46,47^. The research used the plant material (hay, sward and roots) and soils (*Albic Brunic Arenosol,* A, AE and Bsv horizons) sampled 5 and 25 m away from the irrigation ditch in the area of 3 Kamionna (53°50^′^ N; 18°09′ E), Cegielnia (53°53′ N; 18°07′ E) and Podlesie (53°51′ N; 18°08′ E ) quarters, being part of the Czerskie Meadows. In total the research covered 6 soil profiles (a total of 18 plant material samples and 18 soil samples). The soil samples were dried in room temperature and sieved (2 mm). The plant material samples were dried in room temperature and ground. The grain size and the chemical composition of soils are given in Table 1. The flora and phytosociology studies^46^ have shown that in that area there formed plant communities which, in terms of phytosociology, are different subtypes of ryegrass meadows the class *Molinio – Arrherenatheretea* and the order *Arrherenatheretalia.* The variation in precipitation and the mean annual temperature are provided in Fig. 1.

**Table 1:** Basic parameters of soils and grain size composition.

Horizon		TOC (g kg^-1^)	Nt (g kg^-1^)	pH	0.2–0.05 mm (%)	0.05–0.002 mm (%)	< 0.002 mm (%)
A	Mean	16.1	1.19	–	91.92	8.06	0.02
	Min	7.8	0.40	5.3	88.53	6.26	0.02
	Max	26.8	2.34	6.9	93.72	11.45	0.02
	SE*	9.1	0.83	–	1.88	1.88	–
AE	Mean	51.1	4.36	–	90.36	9.60	0.075
	Min	43.5	3.59	5.90	87.13	7.44	0.02
	Max	64.5	5.50	7.00	92.56	12.92	0.20
	SE	8.0	0.73	–	2.09	2.12	0.084
Bsv	Mean	36.3	2.84	–	89.80	9.62	0.57
	Min	16.3	1.35	6.4	87.23	7.46	0.02
	Max	46.3	4.41	7.0	92.00	11.70	1.04
	SE	13.6	1.28	–	2.30	1.96	0.42

*Standard error.

## Methods

### *For air-dry soil samples the following analyses were made*:

- the content of total organic carbon (TOC) and total nitrogen (Nt). The content of organic carbon and total nitrogen were assayed with the Vario Max CN analyzer provided by Elementar (Germany). The content of TOC and Nt was expressed in g kg^-1^ of d.w. of soil^48^;
- pH – in the suspension of distilled water and soil with the pH-meter MultiCal pH 540 GLP WTW^49^;
- grain size composition was determined applying the areometric method^50^;

#### Extraction of phenolic compounds from the samples of plant material and soils

**Figure 2:**
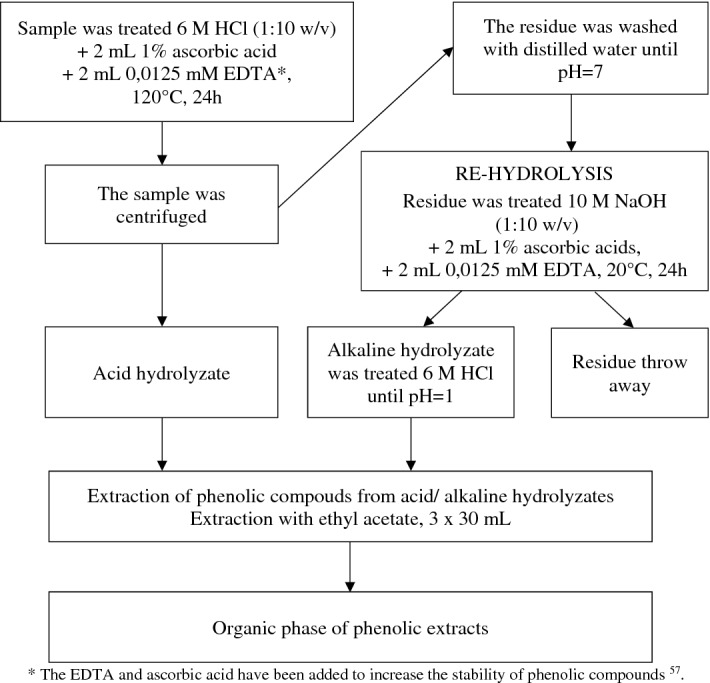
Diagram of the procedure of extracting phenolic compounds from plant material and from soil.

The phenolic compounds contained in air-dry ground samples of plant material and soil samples were extracted compliant with the diagram presented in Fig. 2. The results of the content of phenolic compounds constituted their total contents assayed in the extracts following acid and alkaline hydrolysis.

#### Extraction of phenolic compounds from the fraction of humic and fulvic acids

**Figure 3:**
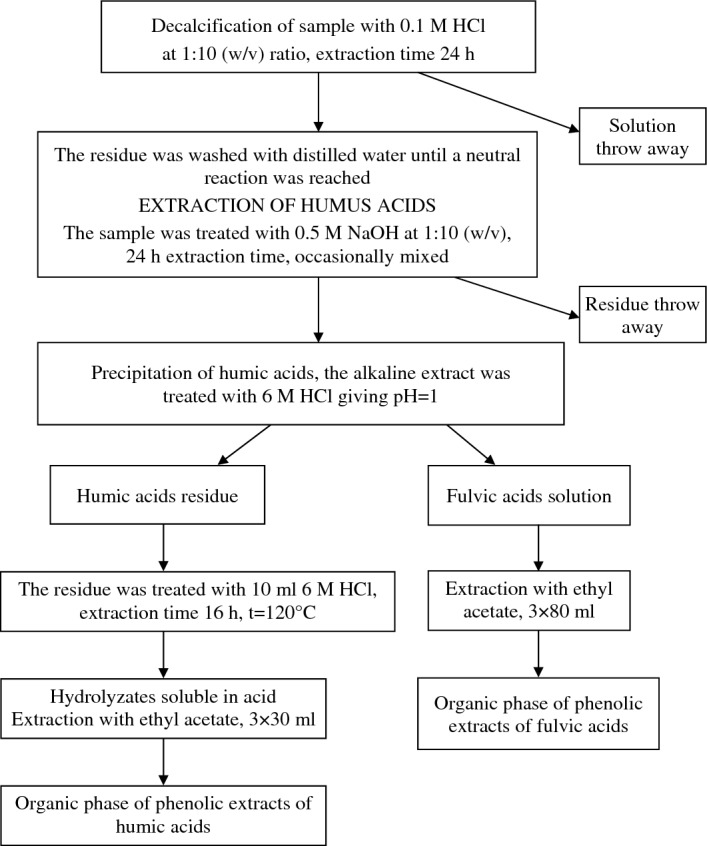
Diagram of the procedure of extracting phenolic compounds from humic and fulvic acids.

The phenolic compounds were extracted from the fraction of fulvic acids and from hydrolysates of the fraction of humic acids compliant with the diagram in Fig. 3.

#### Qualitative and quantitative analysis of extracts containing phenolic compounds

The qualitative and quantitative composition of phenolic compounds contained in the extracts from hydrolysates from the samples of the plant material, the samples of soils and the fractions of humic and fulvic acids were assayed using the Perkin-Elmer 200 HPLC system with a DAD detector. Analytic column Bionacom Velocity STR with 5 μm in particle diameter and the size 250 × 4.6 mm I.D. was used.

The mobile phase consisted of:

$${\text{eluent}}\;{\text{A}}:{\text{ H}}_{{2}} {\text{O}}:{\text{CH}}_{{3}} {\text{CN}}:{\text{CH}}_{{3}} {\text{COOH }}\left( {{88}.{5}:{1}0:{1}.{5}{-}{\text{ratio}}\;{\text{in}}\;\% {\text{V}}} \right),$$

$${\text{eluent B}}:{\text{CH}}_{{3}} {\text{CN}}.$$

The samples for analysis were prepared by combining 0.5 mL filtered, applying the nylon injection filter (φ = 0.45 µm), extract of phenolic compounds with 0.5 mL of eluent A. The injection volume was 10 µL. A gradient separation program was used at the flow rate of 1.3 mL min^-1^. The initial composition of the mobile phase included 100% of eluent A. The content of eluent B was growing linearly from the 14^th^ minute of the analysis and it reached 10% in 36 min, to decrease, reaching at the final stage of the analysis, namely in the 42nd minute, 0%. The detection was made at the wavelength of 280 nm. Phenolic compounds were identified from the pattern of the chromatogram for the reference solution of phenolic compounds the list of which is provided in Table 2.

**Table 2:**
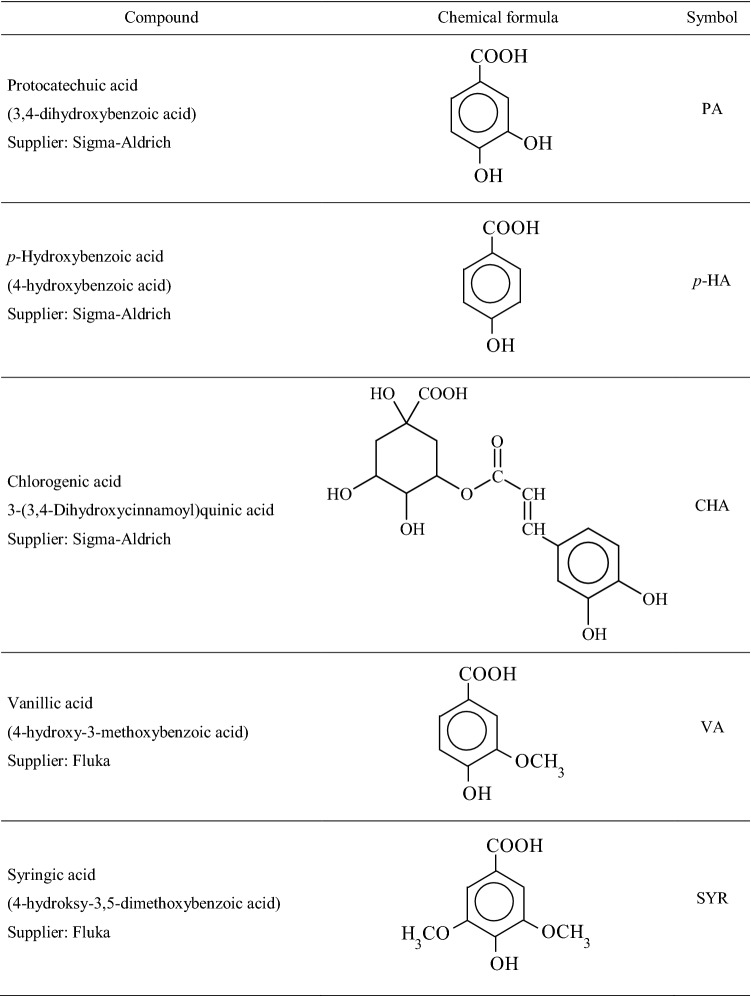

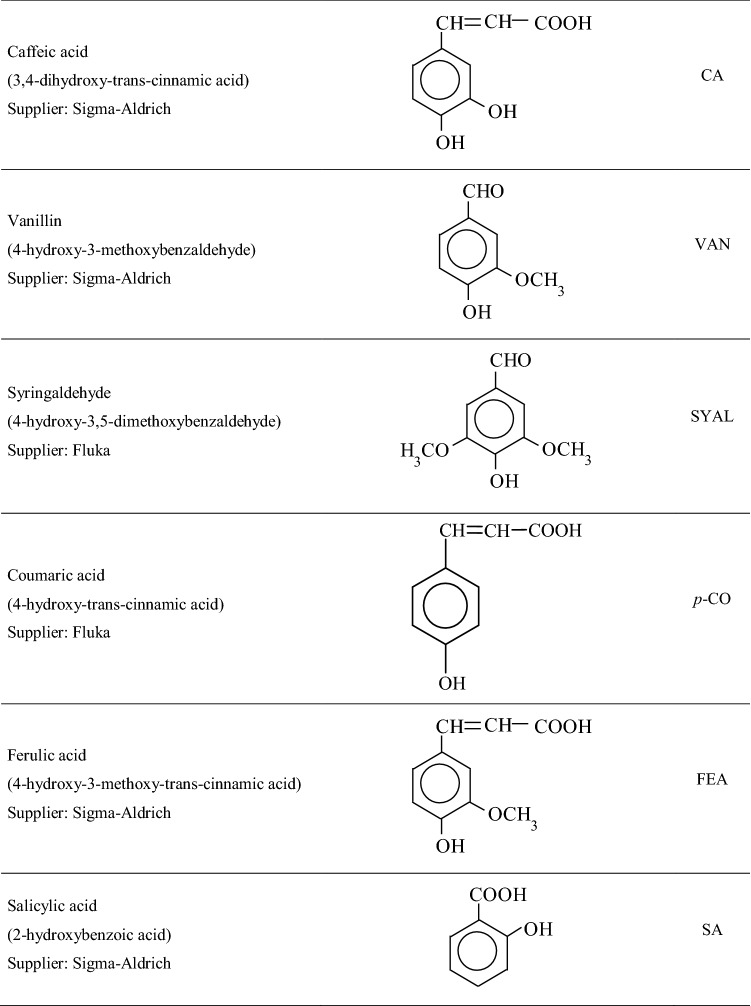
List of the phenolic compounds analysed.

The quantitative analysis of the phenolic compounds identified was performed using the reference curves of the dependence of the peak area on the concentration of phenolic compound (μg mL^-1^). The HPLC analysis of reference solutions was performed as for the phenolic extracts.

With the contents of phenolic compounds, the following were assayed:

- V—total content of vanillin and vanillic acid (VAN + VA),
- S—total content of syringaldehyde and syringic acid (SYAL + SYR),
- C—total content of ferulic acid and caffeic acid (FEA + CA),

and the following parameters were calculated:

- V + S + C—sum of phenols of vanillyl, syringyl and cinnamyl type,
- V:S:C—ratio of the share of respective compounds (modification of the method described in^39^).

The results were statistically verified by determining the following: mean, maximum and minimum, standard error (SE); with significance of the differences of the values of the parameters depending on the plant material sample type (hay, sward and roots), genetic horizon of the soil samples (A, AE and Bsv) with the Kruskal–Wallis test and median test and U Mann–Whitney test, at the level of significance of p = 0.05. For statistical calculations the Excel spreadsheet and Statistica MS 2012 package were applied.

## Results and discussion

### The content of phenolic compounds in the samples of plant material, soils and humus acids

The aldehydes and phenolic acids isolated from the material sampled provide important information on the pattern of organic matter humification processes. As reported by some authors^37,44,51,54^, the content of phenolic compounds depends on the degree of organic matter composition and the type of plant residue subject to decomposition.

**Figure 4:**
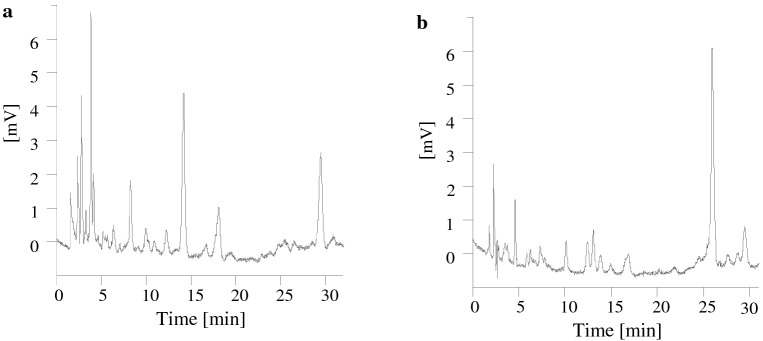
Selected chromatograms of phenolic compounds contained in soil sample extracts (**a)** following the acid hydrolysis, (**b)** following the re-hydrolysis.

In the extracts from the hydrolysates of the samples of the plant material and the soils studied in this paper, 11 phenolic compounds have been identified, and so the research material did not differ in terms of quality. The selected chromatograms of phenolic compounds contained in the extracts from the samples of soil following the acid hydrolysis and following re-hydrolysis presented in Fig. 4. However, significant differences were found in the content of phenolic compounds depending on the material. Most phenolic compounds have been identified in the extracts from plant material hydrolysates (hay > sward > roots). The next one in terms of the content of phenolic compounds was soil (A > AE > Bsv horizon), and the lowest content of aldehydes and phenolic acids was identified in the hydrolysates of humic acids.

In the extracts from the hydrolysates from the plant material samples of all the phenolic compounds the highest amounts of ferulic (FEA), *p*-coumaric acid (*p*-CO) and chlorogenic acids (CHA) were isolated (Table 3).

**Table 3:** Content of phenolic compounds in plant material and in soil (μg g^-1^).

	Mean	Min	Max	SE*	Mean	Min	Max	SE	Mean	Min	Max	SE
	Hay				Sward				Roots			
PA	172.26	129.57	255.17	8.25	153.17	56.95	249.63	15.09	121.28	65.43	202.99	10.54
*p*-HA	288.00	149.97	545.74	32.86	362.70	164.60	673.71	39.10	103.75	18.54	210.68	17.37
CHA	491.98	364.26	751.58	29.98	511.35	272.26	976.76	57.45	306.31	131.29	619.58	35.92
VA	230.92	138.04	315.44	13.19	147.73	102.17	201.02	6.86	137.82	74.69	236.43	11.86
SYR	165.57	92.66	248.63	11.49	159.78	70.60	437.29	29.12	135.61	66.53	247.09	13.66
CA	208.05	117.94	340.56	17.49	213.56	109.80	441.60	25.42	210.19	105.42	374.70	20.47
VAN	331.31	174.83	433.60	18.79	357.84	213.06	596.11	28.05	185.88	87.22	334.31	17.24
SYAL	361.80	197.18	548.79	32.65	201.71	115.82	404.42	21.80	179.62	103.34	312.51	15.26
*p*-CO	1252.88	1028.04	1520.24	37.69	920.53	651.81	1181.84	33.30	934.48	451.57	1251.69	70.29
FEA	2088.08	1446.21	2648.69	96.81	1129.96	666.95	1509.93	57.51	807.30	671.14	1130.93	32.57
SA	146.06	78.42	300.73	15.60	103.57	57.38	206.20	11.42	82.14	15.24	197.24	14.10
Sum	5736.45	5121.81	6375.13	95.71	4260.74	3647.83	4615.91	76.16	3204.72	2542.40	3745.83	115.93

	Soil, horizons A				Soil, horizons AE				Soil, horizons Bsv			
PA	41.84	22.86	64.78	2.86	33.77	18.73	51.65	2.56	23.39	9.70	44.23	2.56
*p*-HA	86.69	48.47	117.51	5.10	111.76	31.42	188.57	14.56	54.71	35.30	76.51	2.97
CHA	135.56	84.36	169.51	5.70	88.82	41.44	132.89	6.11	111.87	36.86	200.38	13.44
VA	53.18	41.53	67.44	1.65	35.44	20.67	56.89	2.25	15.87	8.60	27.38	1.31
SYR	38.81	15.64	84.79	4.78	35.14	17.76	49.37	2.27	22.53	8.36	48.59	2.98
CA	71.23	45.10	102.83	4.15	81.08	42.92	121.02	5.02	36.16	22.01	51.89	2.43
VAN	28.24	17.91	37.82	1.18	34.18	18.58	54.88	2.76	24.87	15.58	39.97	1.56
SYAL	33.63	21.70	46.90	1.78	38.95	14.12	67.63	3.31	24.38	15.59	41.29	1.61
*p*-CO	48.44	14.11	116.23	8.31	20.11	9.41	33.90	1.99	13.75	9.78	21.67	0.69
FEA	103.46	33.57	164.59	10.47	70.69	14.08	133.38	8.12	52.66	19.98	74.58	3.75
SA	25.95	9.95	54.79	2.87	17.97	10.84	32.29	1.46	21.23	12.79	29.29	1.29
Sum	667.02	470.32	854.26	25.76	567.91	497.72	674.05	11.87	401.43	293.22	568.15	19.04

*Standard error.

In the extracts, there were assayed, on average, from 807.30 (roots) to 2088.08 (hay) μg g^-1^ d.w. of ferulic acid, from 920.53 (sward) to 1252.88 (hay) μg g^−1^ d.w. of *p*-coumaric acid and from 306.31 (roots) to 511.35 (sward) μg g^-1^ d.w. of chlorogenic acid. Besides there were assayed high amounts of vanillyl aldehyde (VAN) from 185.88 (roots) to 357.84 μg g^-1^ d.w. (sward) and syringyl aldehyde (SYAL) from 179.6 2 (roots) to 361.80 μg g^-1^ d.w. (hay).

As reported by Mika et al.^9^ and Kovaleva and Kovalev^37^, the characteristic quality of meadow vegetation is the dominant share in the structure of *p*-coumaric and ferulic acids, as compared with the content of the other phenolic compounds. Mika et al.^9^ for grass *Dactylis glomerata* recorded the highest amounts of caffeic (163.4 μg g^-1^ d.w.), ferulic (144.9 μg g^-1^ d.w.) and *p*-coumaric (46.5 μg g^-1^d.w.) acids. The contents of the other phenolic compounds analysed ranged from 2.3 to 12.5 μg g^-1^ d.w. The grass species *Festuca rubra* contained high amounts of caffeic (311.5 μg g^-1^ d.w.), ferulic (251.8 μg g^-1^ d.w.), chlorogenic (172.0 μg g^-1^ d.w.) and *p*-coumaric (170.7 μg g^-1^ d.w.) acids. The content of vanillyl aldehyde was 19.3 μg g^-1^ d.w., protocatechuic acid – 24.1 μg g^-1^ d.w., while *p*-hydroxybenzoic acid – 17.0 μg g^-1^ d.w. The contents of the other phenolic compounds ranged from 2.4 to 15.1 μg g^-1^ d.w. The grass species *Bromus*, as reported by the above Authors, showed the highest, of all the compounds analysed, contents of chlorogenic (126.2 μg g^-1^ d.w.), caffeic (77.7 μg g^-1^ d.w.), ferulic (74.3 μg g^-1^ d.w.), *p*-coumaric (26.7 μg g^-1^ d.w.) and protocatechuic acids (11.2 μg g^-1^ d.w.).

The data presented in the paper also coincides with the results of research of the chemical composition performed by Sterbova et al.^52^ in the grass species of orchard grass *(Dactylis glomerata)*, red fescue *(Festuca rubra)* and bromes *(Bromus)*. The grass species *Festuca* and *Bromus* contained the highest amounts of ferulic (895–1533 μg g^-1^ d.w.), caffeic (617–2561 μg g^-1^ d.w.) and chlorogenic acids (326–2076 μg g^-1^ d.w.). The grass species *Dactylis glomerata* recorded the highest, of all the phenolic compounds analysed, amounts of caffeic (348 μg g^-1^ d.w.) and ferulic acids (110 μg g^-1^ d.w).

Based on own results (Table 3), as well as on the results by Mika et al.^9^ and Sterbova et al.^52^, one must refer to *p*-coumaric, ferulic and chlorogenic acids as dominating in terms of quantity in meadow vegetation samples. Interestingly, one of the factors determining the content of phenolic compounds in plants and in soil are hydrological conditions^35^. The research reported in this paper concern the areas of the temperate climate zone. However, as provided in the methodology section, the meadows were irrigated, and so they showed a high moisture, which could have increased dissolved phenolic compounds leaching deep down the soil profile. On the other hand, as reported in literature^35^, a high content of phenolic compounds in the soil solution decreases the soil organic matter decomposition intensity. The limited intensity of SOM decomposition can result in carbon sequestration and in lowered carbon dioxide emissions.

The chemical composition of the plants affects the chemical composition of organic matter of soils and humus substances. In the extracts from soil hydrolysates, just like in the extracts from the hydrolysates of the plant material, there were identified high contents of chlorogenic acid (CHA) from 88.82 (AE horizon) to 135.56 μg g^-1^ d.w. (A horizon) and ferulic acid (FEA) from 52.66 (Bsv horizon) to 103.46 μg g^-1^ d.w. (A horizon). Besides, in the extracts from soil hydrolysates, high amounts of *p*-hydroxybenzoic acid (*p*-HA), ranging from 54.71 (Bsv horizon) to 111.76 μg g^-1^ d.w. (AE horizon), and caffeic acid from 36.16 (Bsv horizon) to 81.08 μg g^-1^ d.w. (AE horizon), were found. The content of the other acids and aldehydes ranged from 13.75 to 41.84 μg g^-1^ d.w.

In the extracts from fulvic acids the following were dominant: chlorogenic acid (CHA): 5.97 (Bsv horizon) – 32.01 μg g^-1^d.w. (A horizon), ferulic acid (FEA): 4.79 (Bsv horizon) – 24.78 μg g^-1^ d.w. (A horizon) and *p*-coumaric acid (*p*-CO): 4.07 (Bsv horizon) – 48.91 μg g^-1^ d.w (A horizon). The hydrolysates of humic acids showed the highest content of chlorogenic acid. Interestingly, its content in the extracts of humic acids of A horizon was almost sevenfold higher and AE horizon – fivefold higher than the HAs of Bsv horizon. The contents of the other identified phenolic compounds in the extracts of HAs of A horizon fell within the range from 2.85 (*p*-HA) to 7.75 μg g^-1^ d.w. (FEA), AE horizon – from 0.80 (CHA) to 7.81 μg g^-1^ d.w (VA) and Bsv horizon – from 0.58 (CHA) to 2.70 μg g^-1^ d.w (VA).

Generally, lower contents of phenolic compounds in the extracts from plant samples are translated into their lower amounts in the extracts from the hydrolysates from the soil samples and fractions of humus acids (Tables 3 and 4).

**Table 4:** Content of phenolic compounds in fulvic acids (FAs) and humic acids (HAs) (μg g^-1^).

	Mean	Min	Max	SE*	Mean	Min	Max	SE	Mean	Min	Max	SE
	FAs, horizon A				FAs, horizon AE				FAs, horizon Bsv			
PA	8.92	2.05	17.55	1.19	3.76	1.00	10.42	0.79	1.59	0.48	2.87	0.18
*p*-HA	7.00	1.05	14.12	1.03	2.00	0.56	4.64	0.36	1.99	0.65	4.31	0.27
CHA	32.01	17.90	48.23	2.14	21.60	9.29	35.95	2.25	5.97	3.84	10.34	0.51
VA	15.9	10.41	22.15	0.91	5.24	3.02	7.92	0.38	2.49	0.39	9.12	0.65
SYR	11.58	5.50	18.57	0.94	7.26	4.07	12.71	0.63	1.72	0.41	3.13	0.21
CA	13.60	5.49	24.95	1.34	7.78	3.03	11.40	0.57	3.81	0.38	10.87	0.80
VAN	6.42	3.08	12.47	0.64	3.12	2.08	4.96	0.20	1.57	0.18	3.10	0.18
SYAL	10.31	7.04	14.20	0.50	4.61	1.18	10.18	0.77	1.65	0.38	6.95	0.48
*p*-CO	48.91	28.22	70.60	2.59	12.77	6.84	21.02	1.02	4.07	2.04	8.34	0.54
FEA	24.78	11.86	34.55	1.57	8.68	3.03	21.28	1.24	4.79	2.01	8.06	0.39
SA	2.02	1.01	3.32	0.20	2.29	0.43	5.84	0.46	2.11	0.33	4.51	0.25
SUM	181.45	143.92	232.51	5.98	79.10	51.58	99.12	3.29	31.75	17.46	48.63	2.37

	HAs, horizon A				HAs, horizon AE				HAs, horizon Bsv			
PA	5.70	1.28	14.60	1.04	4.25	0.85	8.70	0.62	1.22	0.34	2.05	0.12
*p*-HA	2.85	0.60	6.11	0.39	1.67	1.02	3.03	0.14	1.37	0.44	3.17	0.20
CHA	33.16	6.83	92.63	6.81	22.88	9.39	55.97	3.44	4.86	0.74	10.55	0.85
VA	5.66	3.91	8.16	0.35	7.81	3.79	10.04	0.46	2.70	0.26	9.85	0.73
SYR	4.20	2.06	6.19	0.34	2.87	0.55	7.46	0.58	1.81	0.42	3.44	0.26
CA	4.69	3.19	8.23	0.37	7.39	3.31	11.23	0.56	1.74	0.54	3.61	0.26
VAN	3.19	1.26	4.49	0.24	2.80	1.01	4.97	0.29	1.42	0.21	4.33	0.29
SYAL	4.74	2.16	10.34	0.64	2.71	0.37	6.08	0.44	1.85	0.65	3.84	0.27
*p*-CO	6.36	0.53	18.60	1.46	0.80	0.26	1.55	0.09	0.58	0.16	1.01	0.07
FEA	7.75	3.60	14.03	0.79	1.28	0.33	3.63	0.24	1.26	0.97	1.85	0.06
SA	4.46	0.88	8.81	0.65	1.13	0.42	2.55	0.16	1.08	0.47	1.45	0.08
SUM	82.77	53.02	147.47	7.71	55.6	30.22	97.31	5.14	19.50	12.94	26.48	1.12

*Standard error.

The decreasing contents of phenolic compounds deep down the soil profile are worth noting. Similar dependencies were recorded for the forest soil samples reported by Banach-Szott and Debska^40^, Debska and Banach-Szott^53^. The decreasing contents of phenolic compounds with the sampling depth, as stressed by the Authors, demonstrate that the degree of oxidation (decomposition) of lignins increases with an increase in the degree of organic matter humification.

Interestingly, the content of phenolic compounds in the extracts from hydrolysates from the fraction of HAs was lower than the average contents of phenolic compounds in the extracts from FAs fraction. Fulvic and humic acids, due to genetic conditions, show the same qualitative composition, however, FAs, as compared with HAs, reveal a lower degree of the condensation of aromatic nucleus and so they include more simple aromatic structures, which can be seen from higher contents of phenolic compounds in FAs fractions, as compared with HAs fractions (Table 4).

### Lignin decomposition parameters

Aldehydes and phenolic acids, released from lignins in a form of vanillyl compounds derived from coniferyl alcohol, syringyl compounds – from sinapic alcohol and cinnamyl compounds – from coumaryl alcohol, occur in lignins in specific ratios and depend on the type of organic material. As reported Banach-Szott and Debska^40^ as well as by Kovaleva and Kovalev^37^, the key lignin structure component in conifers are vanillyl compounds and a low content of syringyl compounds, whereas the lignins of deciduous trees contain high and approximately equal amounts of vanillyl and syringyl compounds. In pine needles the ratio V:S:C is 3:0:1, in birch leaves 3:4:1, whereas in the aboveground part of graminaceous plants of subalpine meadows – 1:1:6^37^.

**Figure 5:**
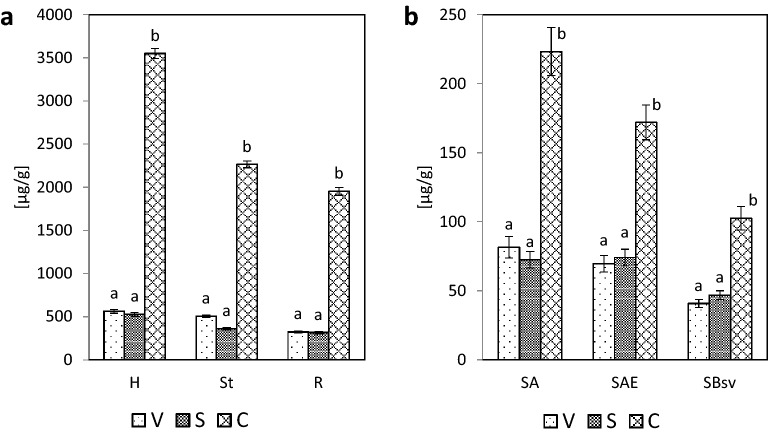
Content of vanillyl (V), syringyl (S) and cinnamyl (C) compounds: **(a)** in the hydrolysates of plant material (H, St, R), **(b)** in soil (SA, SAE, SBsv).

**Figure 6:**
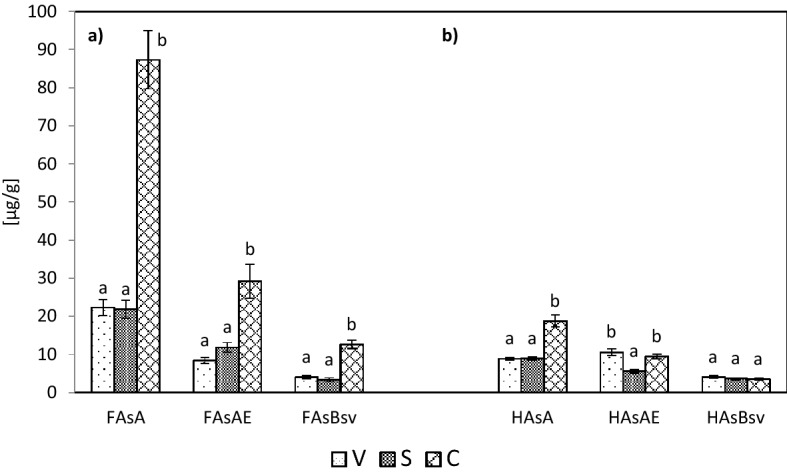
Content of vanillyl (V), syringyl (S) and cinnamyl (C) compounds: (**a)** in the fraction of FAs (FAsA, FAsAE, FAsBsv) and (**b**) in the hydrolysates of HAs fraction (HAsA, HAsAE, HAsBsv).

**Figure 7:**
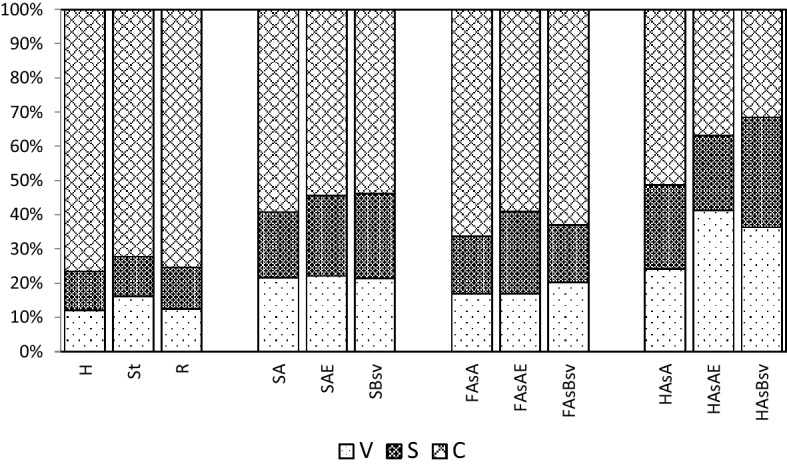
Share of vanillyl (V), syringyl (S) and cinnamyl (C) compounds in V + S + C in the hydrolysates of plant material (H, St, R), in soil (SA, SAE, SBsv), in the fraction of FAs (FAsA, FAsAE, FAsBsv) and in the hydrolysates of HAs fraction (HAsA, HAsAE, HAsBsv).

The dependencies provided in Figs. 5 and 6 show that in the extracts from hydrolysates derived from the samples of plant material (hay, sward and roots), soils and the fractions of fulvic acids and the hydrolysates of the fractions of humic acids of A horizon, the highest mean contents were found for cinnamyl compounds, as compared with the mean contents of vanillyl and syringyl compounds. The V, S and C compounds in the hydrolysates of HAs fractions of AE and Bsv horizons were slightly different. In the extracts from hydrolysates of the fractions of humic acids of AE horizon the contents of cinnamyl compounds were comparable with the contents of vanillyl compounds, while the samples from Bsv horizon were not identified with any significant differences between the V, S and C contents. The high content of cinnamyl compounds in the plant material, soil, fulvic acids and humic acids of A horizon resulted in their high share in V + S + C pool (Fig. 7). In the plant material samples, the share of cinnamyl compounds ranged from 72.31 (roots) to 76.51% (hay), in soil from about 54 (AE and Bsv horizons) to 59.19% (A horizon), in the extracts of FAs – from 59.1 to 66.38% and in the hydrolysates of humic acids – from 31.51 in Bsv horizon to 51.38% in A horizon. The V:S:C ratio for the samples of the plant material changed from 1:1:6 to 1:1:9, in the hydrolysates from the samples of soils, in general, it ranged from 1:1:2.5 to 1:1:3.

The dependencies definitely coincide with the results reported by Kovaleva and Kovalev^37^ pointing to the dominant shares of phenolic compounds type cinnamyl (of *p*-coumaric acid, ferulic and caffeic acids) in the lignins of graminaceous plants.

As reported in literature^39,40,44,53–56^, the quantitative composition of phenolic compounds depends not only on the type of the research material but also on the degree of the decomposition of the organic matter of soil. As seen from the dependencies presented earlier (Fig. 7), the humification process of organic matter of meadow vegetation is related to an increase in the share of vanillyl and syringyl compounds and a decrease in the share of cinnamyl compounds. One must stress, however, that the ratios of vanillyl and syringyl compounds, in general, do not undergo changes (Table 5).

**Table 5:** Ratio of the share of vanillyl (V), syringyl (S) and cinnamyl (C) compounds – V:S:C in hydrolysates of plant material (H, St, R), soil (SA, SAE, SBsv), fraction of FAs (FAsA, FAsAE, FAsBsv) and fraction of HAs (HAsA, HAsAE, HAsBsv).

Sample	H	St	R	SA	SAE	SBsv
V:S:C	1:1:7	1.5:1:6	1:1:6	1:1:3	1:1:2.5	1:1:3

Sample	FAsA	FAsAE	FAsBsv	HAsA	HAsAE	HAsBsv
V:S:C	1:1:4	1:1:3.5	1:1:3.5	1:1:2	2:1:1.5	1:1:1

An increase in the share of vanillyl compounds and a decrease in the share of cinnamyl compounds in the total pool of the content of S, V and C together with an increase in the degree of organic matter decomposition were also observed by Debska and Banach-Szott^53^ in forest soils in the oak and spruce stands. Kovaleva and Kovalev^37^, investigating the lignin plant material decomposition processes in the soils of alpine and subalpine meadows, observed an increase in the share of both vanillyl and syringyl compounds.

The lowest share of cinnamyl compounds was found in the HAs hydrolysates, Bsv horizon – 1:1:1 (Table 5). Also, Kovaleva and Kovalev^37^, for the humic acids of subalpine soils, recorded the shares of vanillyl, syringyl and cinnamyl compounds in the ratio 1:1:1. In the molecules of fulvic acids of the meadow soils under study the ratios of V:S:C compounds was 1:1:3.5 (AE and Bsv horizons) and 1:1:4 (A horizon). As commonly known, fulvic acids, as compared to humic acids, show a lower degree of “maturity”; hence the ratios of the compounds are closer to the V:S:C ratio values recorded for the hydrolysates of soils than those recorded for humic acids.

The advantage of cinnamyl compounds in the extracts of fulvic and humic acids in A and AE horizons shows a definite effect of the chemical composition of materials undergoing decomposition on the properties of the humus substances produced.

Essential information on the pattern of the process of humification is also provided by the parameter of the sum of vanillyl, syringyl and cinnamyl compounds V + S + C applied as a measure of undisturbed uncondensed lignin structures^37,39^.

**Figure 8:**
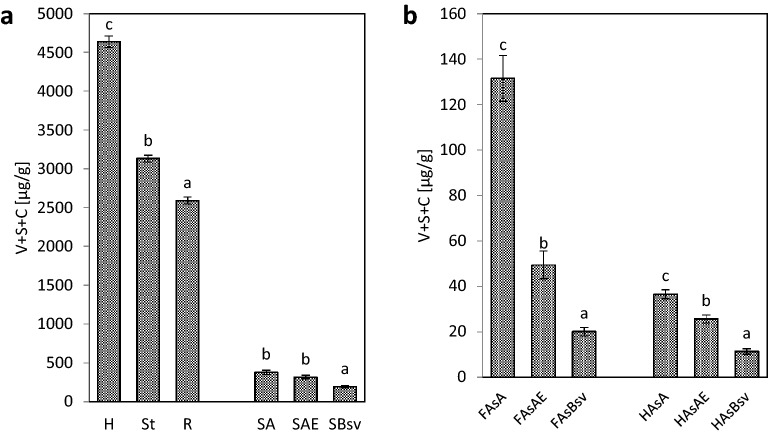
Sum of the content of vanillyl (V), syringyl (S) and cinnamyl (C) compounds (V + S + C): (**a)** in the hydrolysates of plant material (H, St, R) and in soil (SA, SAE, SBsv), **(b)** in the fraction of FAs (FAsA, FAsAE, FAsBsv) and the hydrolysates of HAs fraction (HAsA, HAsAE, HAsBsv).

The values of the parameter V + S + C (Fig. 8) were found in the decreasing order: plant > soil > fractions of fulvic acids > fractions of humic acids and they were decreasing deep down the soil profile both for the extracts from the hydrolysates of soils and from the fractions of FAs and HAs. The values of the parameter V + S + C for the plant material ranged from 4638.6 (hay) to 2590.9 μg g^-1^ (roots) and for the hydrolysates from soil samples – from 376.99 (A horizon) to 190.22 μg g^-1^ (Bsv horizon), fulvic acids – from 131.50 to 20.10 μg g^-1^ and humic acids – in the range from 36.59 to 11.36 μg g^-1^. The decreasing value of the parameter V + S + C together with the soil profile depth was also recorded by Banach-Szott and Debska^39,40^, Debska and Banach-Szott^53^ for humus acids isolated from the samples of forest soils, as well as by Kovalev and Kovaleva^36^ and Kovaleva and Kovalev^37^ for the samples of the soils of forest and meadow ecosystems.

## Conclusions

The content of phenolic compounds was decreasing in the following order:

hay > sward > roots > horizon A soil > AE horizon soil > Bsv horizon soil > FAs of A horizon > FAs of AE horizon > FAs of Bsv horizon > HAs of A horizon > HAs of AE horizon > HAs of Bsv horizon.

Of all the phenolic compounds identified in the plant material (hay, sward, roots) and the soil samples, the following were dominant: ferulic, p-coumaric and chlorogenic acids the share of which was 6.8–36.7% (plant material) and 3.8 to 35.7% (soil), respectively.

A comparison of the results of the present research with the literature data demonstrates that the ratios of phenolic compounds in plant materials are characteristic and so they are plant species specific; however the ratios do not depend on the climate conditions. The meadow vegetation showed a considerable share of cinnamyl compounds (C) as compared with the shares of vanillyl (V) and syringyl (S) compounds. The ratios V:S:C ranged from 1:1:6 to 1:1:9. During the process of humification of meadow vegetation the share of cinnamyl compounds decreases, while the share of syringyl and vanillyl compounds—increases, at the same time maintaining the ratio V:S similar to 1:1.

The parametr V:S:C found for the extracts from soil hydrolysates and humus acids is the parameter which indicates the type of the plant material undergoing the processes of decomposition.
